# Localization of Presynaptic Plasticity Mechanisms Enables Functional Independence of Synaptic and Ectopic Transmission in the Cerebellum

**DOI:** 10.1155/2015/602356

**Published:** 2015-06-10

**Authors:** Katharine L. Dobson, Tomas C. Bellamy

**Affiliations:** School of Life Sciences, University of Nottingham Medical School, Nottingham NG7 2UH, UK

## Abstract

In the cerebellar molecular layer parallel fibre terminals release glutamate from both the active zone and from extrasynaptic “ectopic” sites. Ectopic release mediates transmission to the Bergmann glia that ensheathe the synapse, activating Ca^2+^-permeable AMPA receptors and glutamate transporters. Parallel fibre terminals exhibit several forms of presynaptic plasticity, including cAMP-dependent long-term potentiation and endocannabinoid-dependent long-term depression, but it is not known whether these presynaptic forms of long-term plasticity also influence ectopic transmission to Bergmann glia. Stimulation of parallel fibre inputs at 16 Hz evoked LTP of synaptic transmission, but LTD of ectopic transmission. Pharmacological activation of adenylyl cyclase by forskolin caused LTP at Purkinje neurons, but only transient potentiation at Bergmann glia, reinforcing the concept that ectopic sites lack the capacity to express sustained cAMP-dependent potentiation. Activation of mGluR1 caused depression of synaptic transmission via retrograde endocannabinoid signalling but had no significant effect at ectopic sites. In contrast, activation of NMDA receptors suppressed both synaptic and ectopic transmission. The results suggest that the signalling mechanisms for presynaptic LTP and retrograde depression by endocannabinoids are restricted to the active zone at parallel fibre synapses, allowing independent modulation of synaptic transmission to Purkinje neurons and ectopic transmission to Bergmann glia.

## 1. Introduction

In the cerebellar cortex, transmission from parallel and climbing fibre terminals to Bergmann glial cells occurs at dedicated release sites that are “ectopic” to the presynaptic active zone [1, 2]. Ectopic release sites express short-term plasticity that resembles the adjacent synaptic sites in some respects but differs in others. At the parallel fibre, paired pulse stimulation causes facilitation of excitatory postsynaptic currents (EPSCs) for the second pulse, a phenomenon attributed to summation of presynaptic calcium levels increasing release probability at the terminal [3]. This is true for both synaptic and ectopic transmission, but ectopic sites show a greater degree of facilitation than synaptic sites (5-fold versus 2-fold [4, 5]). Conversely, the climbing fibre terminal exhibits paired pulse depression, which is attributed to depletion of the readily releasable pool by the initial high-release-probability stimulus. Again, ectopic sites mirror the synaptic response, but with a more profound and longer-lasting depression (0.1-fold versus 0.5-fold [4]).

In addition to these features of short-term plasticity, ectopic release also exhibits characteristic long-term plasticity. Stimulating either parallel or climbing fibres at frequencies greater than ~0.1 Hz leads to a progressive, input-specific depression of transmission [6], which results from depletion of ectopic vesicle pools through an absence of the recycling mechanisms present at synaptic sites [7]. This property of ectopic transmission results in glia receiving a strength of input that is inversely proportional to the average firing rate of the terminal [7].

These features of ectopic and synaptic sites suggest that transmission to Purkinje neurons and Bergmann glia can be, to an extent, operationally independent, in keeping with the differing roles of the cells in cerebellar function. However, several forms of presynaptic plasticity are known at the parallel fibre synapse [8], and if both release sites are present within the same terminal [2], neuron-glial transmission may respond to the same stimulation patterns and so may exhibit the same computational properties as synaptic transmission. Accordingly, glia may share the same forms of presynaptic plasticity as neurons, but differ in postsynaptic forms.

The best characterized form of presynaptic plasticity at the parallel fibre is long-term potentiation (LTP) evoked by brief stimulation at 4–16 Hz [9–13]. This form of LTP depends on an increase in presynaptic cAMP concentration, activation of PKA and Epacs, and phosphorylation of various components of the vesicle docking and fusion machinery to increase release probability [12–14]. Such a brief stimulus would not itself lead to substantial depletion of ectopic pools, and so similar potentiation of ectopic release could also occur and possibly provide a mechanism for reversing the depletion of ectopic pools that leads to depression of neuron-glial transmission.

In addition to presynaptic LTP, presynaptic long-term depression (LTD) has also been described under conditions when PKA is inhibited [15]. This form of plasticity depends on postsynaptic endocannabinoid release and activation of presynaptic CB1 receptors [15, 16]. This retrograde inhibition is also implicated in classical postsynaptic LTD [17] and can be evoked by pharmacological activation of mGluR1 receptors [18–20]. In addition, presynaptic LTD requires activation of NMDA receptors, most likely expressed on molecular layer interneurons, a signalling loop that has also been implicated in presynaptic LTP [15, 21].

In this study we compare synaptic and ectopic transmission in cerebellar slices from juvenile rats during activity-dependent and pharmacological induction of presynaptic LTP and LTD, under stimulation conditions that would not lead to depletion of ectopic vesicle pools. Our goal was to investigate whether the two presynaptic pools were subject to the same plasticity mechanisms. The results indicate that induction and maintenance of presynaptic plasticity is localized to the active zone sites, and ectopic transmission to glia is largely insensitive to the signalling pathways associated with both LTP and retrograde initiation of LTD. This is further evidence for the functional independence of synaptic and ectopic transmission, enabling separate computational rules to govern changes in the connectivity of synaptic and neuron-glial signalling networks. 

## 2. Materials and Methods

### 2.1. Animals

Rats (age 16–20 days) were humanely killed by cervical dislocation. All experiments were performed according to policies on the care and use of laboratory animals of British Home Office and European Community laws. The University of Nottingham Animal Welfare and Ethical Review Body approved the experiments. All efforts were made to minimize animal suffering and reduce the number of animals used.

### 2.2. Cerebellar Slice Preparation

Transverse cerebellar slices (300 *μ*m) were prepared from 16- to 20-day-old Wistar rats of either sex. Animals were humanely killed by cervical dislocation, decapitated, and the cerebellum rapidly excised. Slices were prepared using a vibrating microtome (Leica VT1000S) in chilled, sucrose-based artificial cerebrospinal fluid (aCSF) containing (mM): sucrose (206), KCl (2.8), CaCl_2_ (1), MgCl_2_ (1), MgSO_4_ (2), NaHCO_3_ (26), glucose (10), ascorbic acid (0.4), and NaH_2_PO_4_ (1.25). Slices were allowed to recover for 1 hour at 32°C, with a further 30 min at room temperature, in aCSF containing (mM) NaCl (126), KCl (3), NaH_2_PO_4_ (1.2), NaHCO_3_ (25), glucose (15), MgSO_4_ (2), and CaCl_2_ (2). For recording, slices were transferred to an immersion chamber and perfused with aCSF containing a lower concentration of MgSO_4_ (1 mM). All solutions were continuously bubbled with carbogen (95% O_2_, 5% CO_2_) throughout. For all experiments, the bath solution was supplemented with 20 *μ*M picrotoxin to inhibit GABA_A_ receptors.

### 2.3. Electrophysiology

Borosilicate recording electrodes were manufactured as previously described [6]. Internal solution consisted of (mM) K-gluconate (110), KCl (5), HEPES (50), EGTA (0.05), MgSO_4_ (4), ATP (4), GTP (0.2), phosphocreatine (9), and pH to 7.4 with 1 M KOH. For 16 Hz tetanus and forskolin experiments the internal solution was supplemented with 10 mM BAPTA. Whole-cell voltage clamp recordings were made from Bergmann glia (holding potential −80 mV) and Purkinje neuron (holding potential −70 mV) somata in the Purkinje cell layer. Currents were low pass filtered at 4-5 kHz and sampled at 25 kHz, using Spike2 software (CED, Cambridge, UK). Series resistances ranged from 5 to 15 MΩ and were compensated by >85% in Purkinje neuron recordings but uncompensated in glial recordings.

Parallel fibres were stimulated with a patch electrode (~1-2 MΩ) filled with bath solution and positioned in the molecular layer, connected to an isolated constant current stimulator (5–40 *μ*A, 80 *μ*s; Digitimer, Welwyn Garden City, UK). Stimulus was delivered as a pair of pulses with a 100 ms interval at a frequency of 0.033 Hz. Tetanic stimulation was delivered at 16 Hz (single pulses) for a 15 s period.

Experiments were performed at room temperature.

### 2.4. Materials

CGP 52432 3-[[(3,4-dichlorophenyl)methyl]amino]propyl diethoxymethylphosphinic acid, DPCPX (8-cyclopentyl-1,3-dipropylxanthine), MPPG ((RS)-*α*-methyl-4-phosphonophenylglycine),* S*-DHPG ((*S*)-3,5-dihydroxyphenylglycine), NMDA (*N*-methyl-D-aspartic acid), ODQ (1*H*-[1,2,4]oxadiazolo[4,3-*a*]quinoxalin-1-one), L-NAME (NG-nitro-L-arginine methyl ester hydrochloride), and picrotoxin were purchased from Tocris Bioscience (Bristol, UK). BAPTA (1,2-bis(2-aminophenoxy)ethane-N,N,N′,N′-tetraacetic acid) and SR141716 (rimonabant hydrochloride) were purchased from VWR International (Lutterworth, Leicestershire, UK). Forskolin was purchased from Fisher Scientific (Loughborough, Leicestershire, UK). Stock solutions of SR141716 and DPCPX were prepared in DMSO, with a final DMSO concentration of less than 0.5%. All other drugs were dissolved directly into the bath solution.

### 2.5. Data Analysis

Glial extrasynaptic current (ESC) and neuronal excitatory postsynaptic current (EPSC) traces shown are the average of five sequential recordings at the indicated frequency. Stimulus artefacts are truncated for clarity. Aggregate data are the mean ± s.e.m. of multiple cells, as indicated in figure captions. Statistical significance of normalized data was tested for by one-sample *t*-tests (for comparison to pretreatment amplitude) or unpaired *t*-test (for comparison between two treatments); multiple comparisons were tested using one-way ANOVA followed by Dunnett's test. Differences were considered significant if *P* < 0.05.

## 3. Results

### 3.1. Presynaptic LTP Is Absent at Ectopic Sites

**Figure 1:**
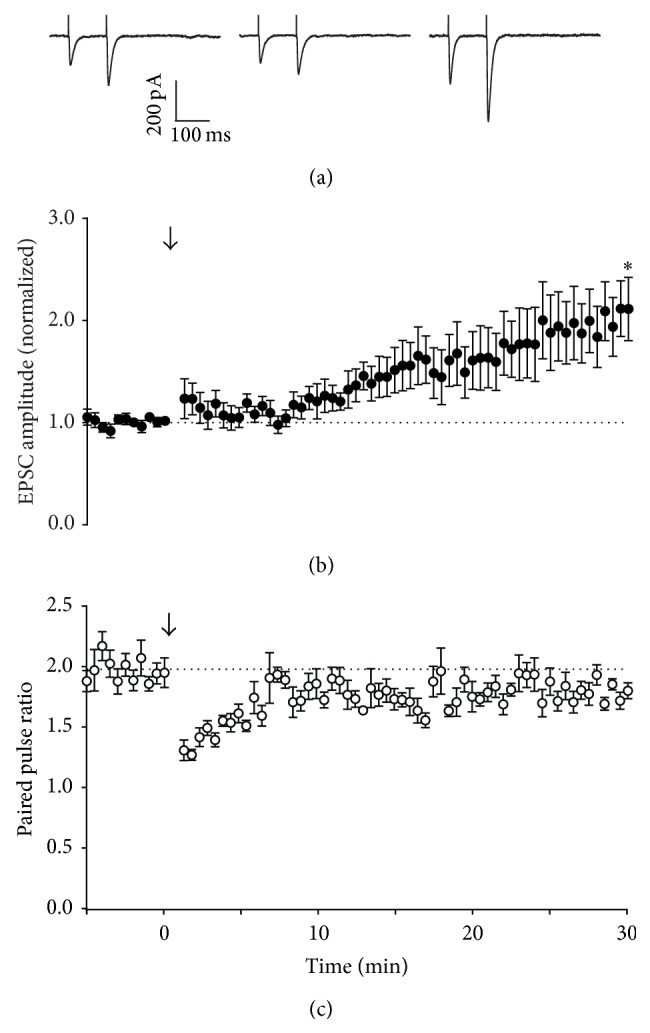
Effect of 16 Hz tetanic stimulation on Purkinje neuron responses. (a) Representative whole-cell recordings of neuronal excitatory postsynaptic currents (EPSC) generated by paired pulse stimulation (100 ms interval) of parallel fibres in transverse cerebellar slices at 0.033 Hz before (first panel), immediately after (second panel), and 30 minutes after (third panel) raised frequency stimulation (RFS, 16 Hz, 15 s). (b) Time course of RFS effect on amplitude of the first pulse in each pair, with RFS delivered at *t* = 0, indicated by the arrow. Data are mean ± s.e.m. from 7 cells. ^*∗*^ *P* = 0.0119, one-sample *t*-test. (c) Time course of RFS effect on paired pulse ratio after tetanic stimulation at *t* = 0. Data are mean ± s.e.m. from 7 cells.

**Figure 2:**
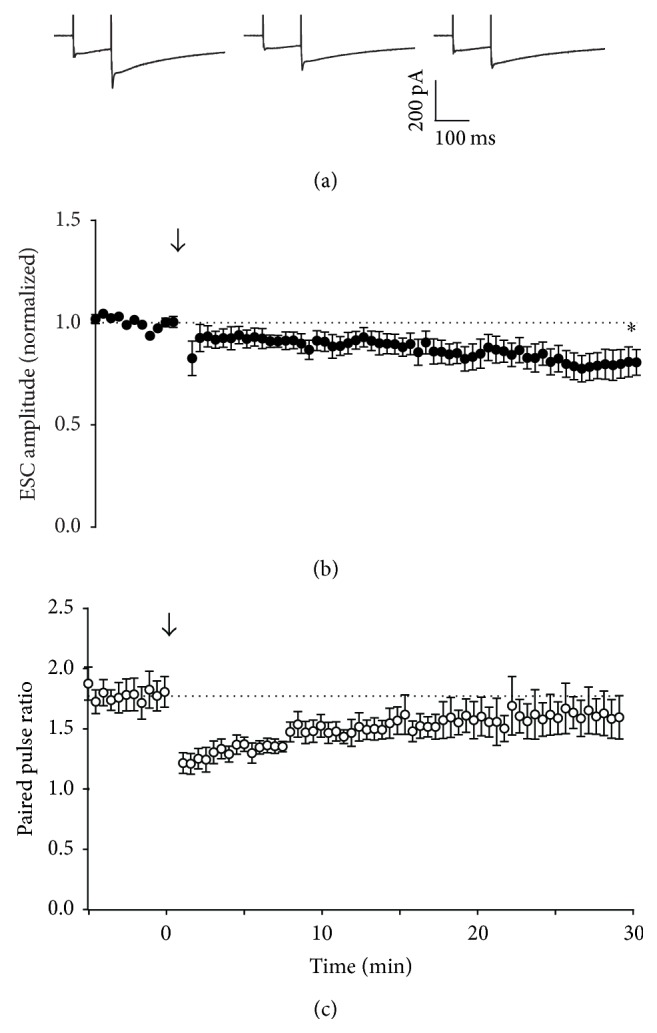
Effect of 16 Hz tetanic stimulation on Bergmann glial responses. (a) Representative whole-cell recordings of glial extrasynaptic currents (ESC) generated by paired pulse stimulation (100 ms interval) of parallel fibres in transverse cerebellar slices at 0.033 Hz before (first panel), immediately after (second panel), and 30 minutes after (third panel) RFS. (b) Time course of RFS effect on amplitude of the first pulse in each pair, with RFS delivered at *t* = 0, indicated by the arrow. Data are mean ± s.e.m. from 6 cells. ^*∗*^ *P* = 0.0177, one-sample *t*-test. (c) Time course of RFS effect on paired pulse ratio after tetanic stimulation at *t* = 0. Data are mean ± s.e.m. from 6 cells.

Brief stimulation of parallel fibres at 4–16 Hz has been reported to evoke long-term potentiation (LTP) of transmission to Purkinje neurons, mediated by activation of cAMP signalling pathways [9, 11, 21]. Consistent with these earlier reports, a 16 Hz, 15 s stimulus evoked LTP of Purkinje neuron EPSCs under our experimental conditions (Figures 1(a) and 1(b)), with an associated decrease in paired pulse ratio (Figure 1(c)), suggestive of an increase in presynaptic release probability. However, the same stimulus applied to parallel fibres while recording from Bergmann glia resulted not in LTP, but LTD, of extrasynaptic currents (ESCs; 0.81 ± 0.06-fold after 30 min, relative to pretetanus amplitude; Figures 2(a) and 2(b)). Curiously, despite exhibiting LTD, paired pulse ratio for Bergmann glial ESCs also decreased after 16 Hz stimulation (to 0.90 ± 0.08-fold; Figure 2(c)).

**Figure 3:**
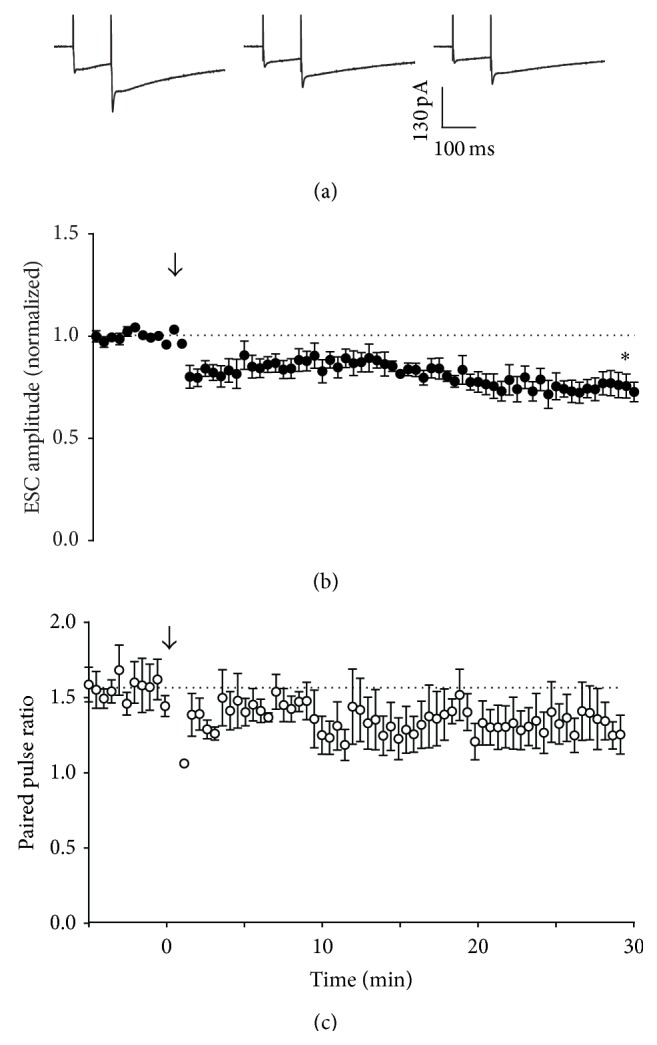
Effect of 16 Hz tetanic stimulation on Bergmann glial responses in the presence of presynaptic receptor antagonists. Slices were preincubated with a cocktail of presynaptic receptor antagonists (2 *μ*M DPCPX, 10 *μ*M CGP 52432, 100 *μ*M MPPG, and 5 *μ*M SR141716) for 10 minutes prior to RFS and then for the 30 minutes following RFS. (a) Representative whole-cell recordings of ESCs generated by paired pulse stimulation (100 ms interval) of parallel fibres in transverse cerebellar slices at 0.033 Hz before (first panel), immediately after (second panel), and 30 minutes after (third panel) raised frequency stimulation (RFS, 16 Hz, 15 s). (b) Time course of RFS effect on amplitude of the first pulse in each pair, with RFS delivered at *t* = 0, indicated by the arrow. Data are mean ± s.e.m. from 4 cells. ^*∗*^ *P* = 0.0192, one-sample *t*-test. (c) Time course of RFS effect on paired pulse ratio after tetanic stimulation at *t* = 0. Data are mean ± s.e.m. from 4 cells.

A previous study by Qiu and Knöpfel [15] reported presynaptic LTD under conditions where signalling via PKA was inhibited, which depended on activation of presynaptic CB1 receptors. To investigate the mechanism of ectopic LTD, we preincubated the slice with a cocktail of presynaptic receptor antagonists (A_1_ adenosine, GABA_B_, group III metabotropic glutamate, and CB1 cannabinoid receptors; see also Figure 3 legend) and repeated the 16 Hz stimulus. The result was indistinguishable from control: LTD of ectopic transmission was evoked despite blockade of presynaptic receptors (0.73 ± 0.05-fold at 30 min, relative to pretetanus amplitude; Figures 3(a) and 3(b)).

**Figure 4:**
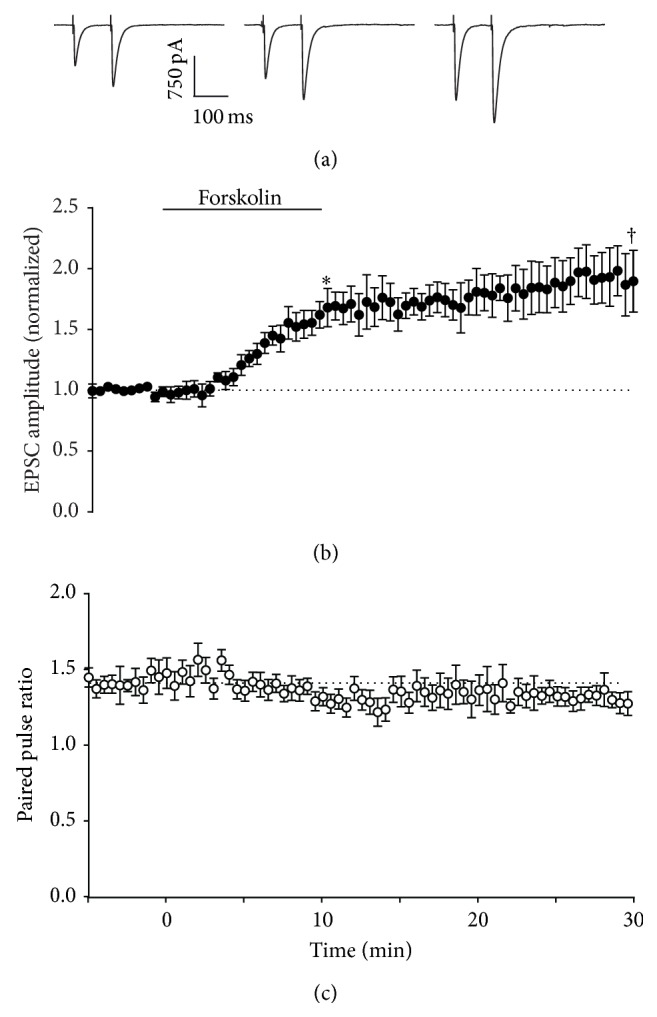
Effect of activation of adenylyl cyclase on Purkinje neuron responses. (a) Representative whole-cell recordings of EPSCs generated by paired pulse stimulation (100 ms interval) of parallel fibres in transverse cerebellar slices at 0.033 Hz before (first panel), after 10 minutes incubation with 50 *μ*M forskolin (second panel), and after 20 minutes of washout (third panel). (b) Time course of 50 *μ*M forskolin effect on amplitude of the first pulse in each pair, with forskolin incubation at the time indicated by the bar. Data are mean ± s.e.m. from 6 cells. ^*∗*^ *P* = 0.0039, ^†^ *P* = 0.0115, one-sample *t*-test. (c) Time course of 50 *μ*M forskolin effect on paired pulse ratio, with forskolin incubation at the time indicated by the bar. Data are mean ± s.e.m. from 6 cells.

**Figure 5:**
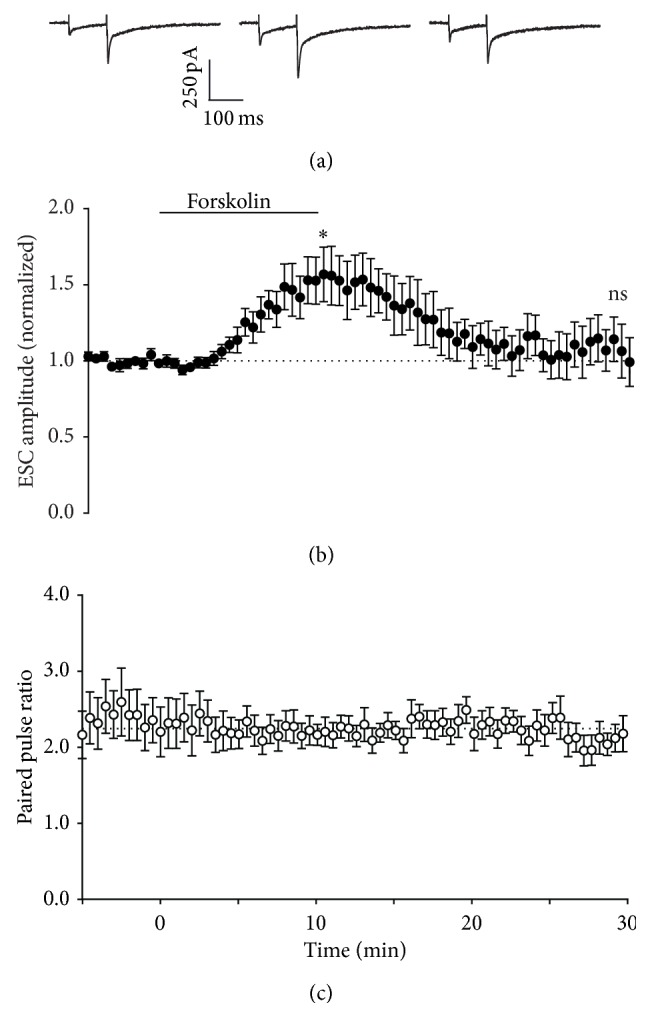
Effect of activation of adenylyl cyclase on Bergmann glial responses. (a) Representative whole-cell recordings of ESCs generated by paired pulse stimulation (100 ms interval) of parallel fibres in transverse cerebellar slices at 0.033 Hz before (first panel), after 10 minutes incubation with 50 *μ*M forskolin (second panel), and after 20 minutes of washout (third panel). (b) Time course of 50 *μ*M forskolin effect on amplitude of the first pulse in each pair, with forskolin incubation at the time indicated by the bar. Data are mean ± s.e.m. from 7 cells. ^*∗*^ *P* = 0.0162, one-sample *t*-test. (c) Time course of 50 *μ*M forskolin effect on paired pulse ratio, with forskolin incubation at the time indicated by the bar. Data are mean ± s.e.m. from 7 cells.

Direct activation of adenylyl cyclase by forskolin is a pharmacological strategy for evoking presynaptic LTP [9], and so we next tested this approach at synaptic and ectopic sites. Forskolin applied for 10 min resulted in LTP of synaptic transmission, with a small decrease in paired pulse ratio (0.92 ± 0.09-fold relative to pretetanus ratio), as previously reported (Figures 4(a), 4(b), and 4(c) [9, 12]). For ectopic transmission to Bergmann glia, forskolin was also effective at inducing potentiation after 10 minutes, but without obvious effect on paired pulse ratio (Figures 5(a), 5(b), and 5(c)). In further contrast to synaptic transmission, washout of forskolin caused a reversal of the potentiation at ectopic sites (Figure 5(b)), with extrasynaptic currents (ESCs) returning to baseline amplitude within 15–20 min. Thus, despite pharmacological activation of cAMP synthesis being sufficient to induce potentiation of ectopic transmission, it appears that mechanisms necessary to stabilize or reinforce the enhancement in transmission in the longer term are absent at these sites.

### 3.2. Retrograde Endocannabinoid Inhibition Is Absent at Ectopic Sites

Another form of plasticity at the parallel fibre synapse that is expressed presynaptically is retrograde activation of CB1 receptors by 2-AG synthesized and released from Purkinje neurons [22]. Initiation of this feedback mechanism is optimal during a confluence of signals: increased Ca^2+^ concentration (due to climbing fibre evoked depolarization), activation of metabotropic glutamate receptors (during tetanic stimulation of parallel fibres), and, potentially, activation of postsynaptic GABA_B_ receptors that synergize with mGluR1 [23]. Stimulation protocols that evoke retrograde inhibition include short-term posttetanic depression [24, 25], and long-term depression during repetitive paired stimulation of parallel and climbing fibres [17]. These protocols also result in depression of ectopic transmission [7], but in this case, depression results from depletion of ectopic pools due to repetitive stimulation, not from retrograde inhibition (because inhibitors of the relevant receptors have no impact on glial depression). Given the failure to prevent 16 Hz LTD with CB1 receptor antagonists, we explored the potential for retrograde endocannabinoid signalling to affect ectopic transmission under conditions where ectopic pools were intact.

**Figure 6:**
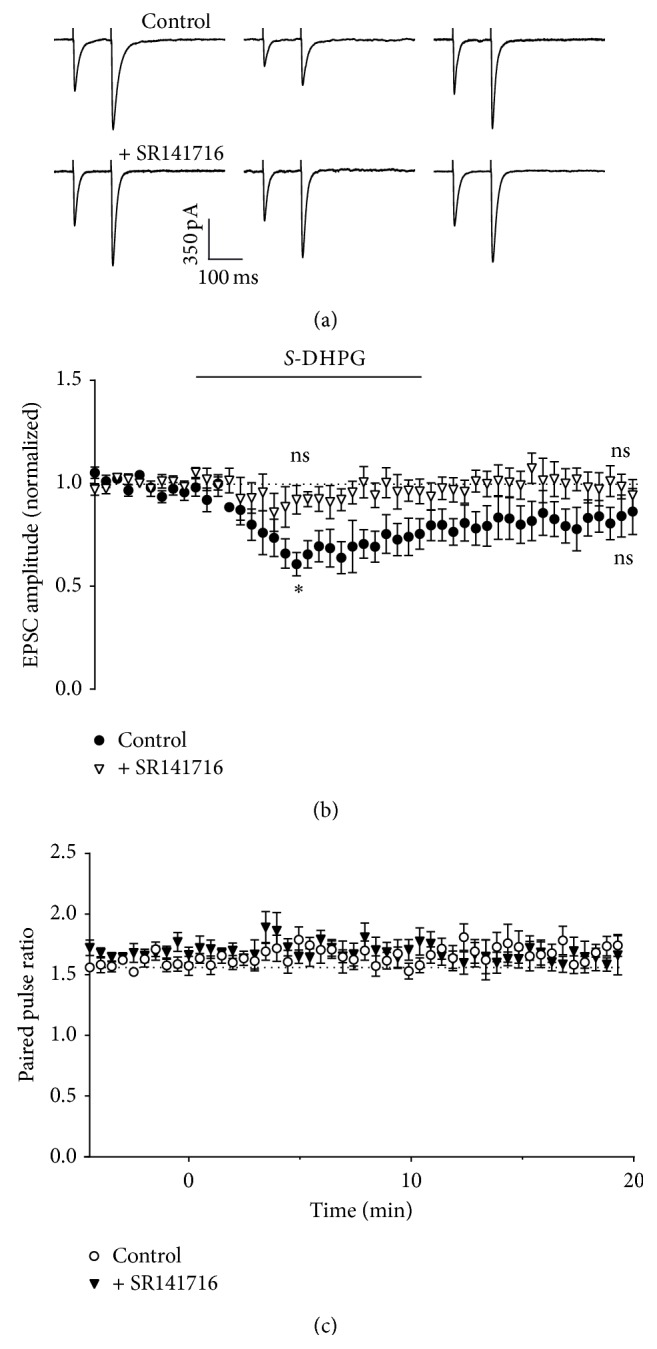
Effect of activation of mGluR1 on Purkinje neuron responses is blocked by antagonism of cannabinoid receptor 1. (a) Representative whole-cell recordings of EPSCs generated by paired pulse stimulation (100 ms interval) of parallel fibres in transverse cerebellar slices at 0.033 Hz before (left hand panels), after 5 minutes incubation with 30 *μ*M DHPG (centre panels), and after 10 minutes of washout (right hand panels). Recordings are shown from cells recorded in the presence of DHPG alone (upper panels) and coapplied with 5 *μ*M SR141716 (lower panels). Cells treated with SR141716 were preincubated with this compound for 5 minutes prior to DHPG addition. (b) Time course of 30 *μ*M DHPG effect on amplitude of the first pulse in each pair, either alone (filled circles) or with 5 *μ*M SR141716 (open triangles), with DHPG incubation at the time indicated by the bar. Data are mean ± s.e.m. from 6 cells for each condition. ^*∗*^ *P* = 0.0014, one-sample *t*-test. (c) Time course of 30 *μ*M DHPG effect on paired pulse ratio, either alone (open circles) or with 5 *μ*M SR141716 (filled triangles), with DHPG incubation at the time indicated by the bar. Data are mean ± s.e.m. from 6 cells for each condition.

**Figure 7:**
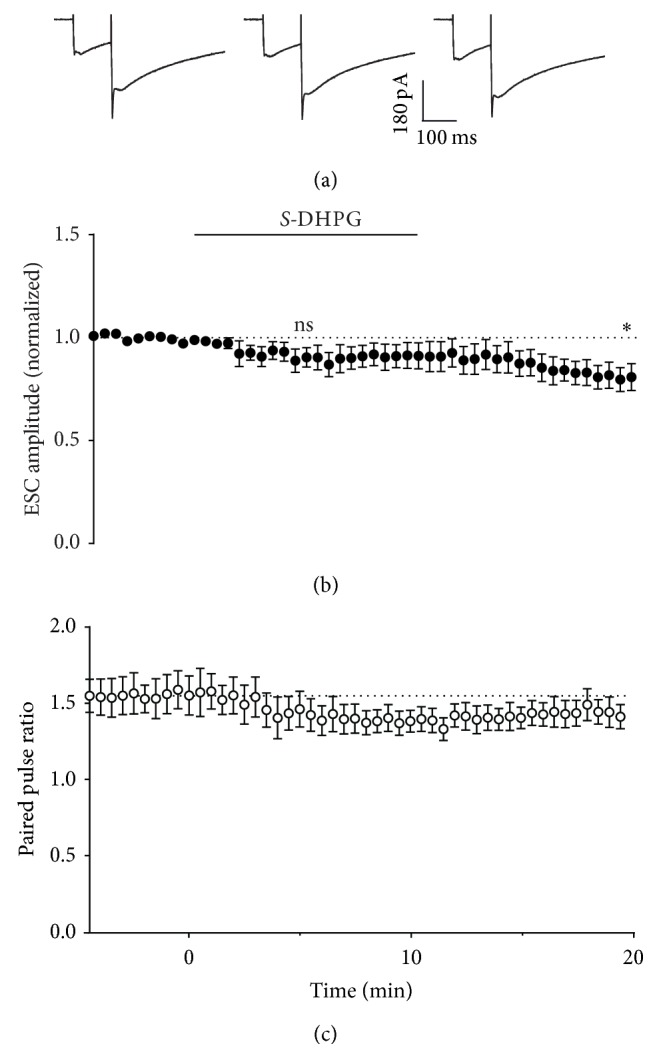
Effect of activation of mGluR1 on Bergmann glial responses. (a) Representative whole-cell recordings ESCs generated by paired pulse stimulation (100 ms interval) of parallel fibres in transverse cerebellar slices at 0.033 Hz before (first panel), after 5 minutes incubation with 30 *μ*M DHPG (second panel), and after 10 minutes of washout (third panel). (b) Time course of 30 *μ*M DHPG effect on amplitude of the first pulse in each pair, with DHPG incubation at the time indicated by the bar. Data are mean ± s.e.m. from 7 cells. ^*∗*^ *P* = 0.0214, one-sample *t*-test. (c) Time course of 30 *μ*M DHPG effect on paired pulse ratio, with DHPG incubation at the time indicated by the bar. Data are mean ± s.e.m. from 7 cells.

Direct pharmacological activation of mGluR1 results in a depression of synaptic transmission, which is blocked by CB1 antagonists [19, 20]. Application of 30 *μ*M *S*-DHPG to the bath solution in our recordings caused a depression of Purkinje neuron EPSCs (to 0.66 ± 0.07-fold of control after 5 minutes; Figures 6(a) and 6(b)). This depression was blocked by the CB1 receptor antagonist SR141716 (Figures 6(a) and 6(b); *P* = 0.0054, unpaired *t*-test at 5 min, DHPG versus DHPG + SR141716). In both cases, paired pulse ratio was largely unaffected by the treatments (1.14 ± 0.07-fold and 0.99 ± 0.03-fold, resp., after 5 minutes; Figure 6(c)). In contrast to these effects on synaptic transmission, the amplitude of ectopic transmission was not significantly affected during the 10 min treatment period (mean amplitude 0.90 ± 0.05-fold of control at 5 minutes; Figures 7(a) and 7(b)). In further contrast to synaptic transmission, which fully recovered from DHPG treatment, ESC amplitude decreased following washout (to 0.81 ± 0.07 of control after 20 minutes), suggesting either a rebound effect or possibly enhanced depletion of ectopic vesicles.

### 3.3. Activation of Interneuron NMDA Receptors Depresses Both Synaptic and Ectopic Sites

LTD of parallel fibre transmission to Purkinje neurons can also be blocked by NMDA receptor antagonists [15, 26]. This discovery was unexpected due to the absence of NMDAR expression in adult Purkinje neurons and has been hypothesized to result from activation of presynaptic receptors on parallel fibre terminals [27] or molecular layer interneurons [15]. The postsynaptic arc of NMDAR-dependent depression appears to require NO synthesis and cGMP signalling [26], but it has also been reported that presynaptic LTD requires activation of both NMDAR and CB1R [15]. In the latter case, the authors speculated that NMDAR activation leads to endocannabinoid release from interneurons, to activate presynaptic CB1R on parallel fibres.

**Figure 8:**
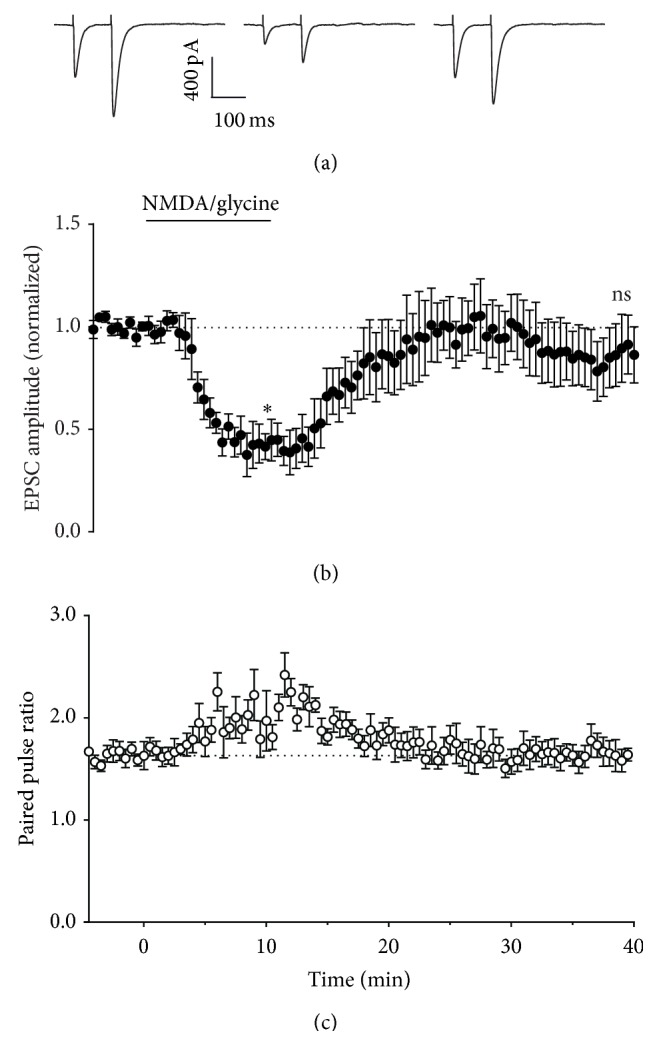
Effect of activation of NMDA receptors on Purkinje neuron responses. (a) Representative whole-cell recordings of EPSCs generated by paired pulse stimulation (100 ms interval) of parallel fibres in transverse cerebellar slices at 0.033 Hz before (first panel), after 10 minutes incubation with 30 *μ*M NMDA and 10 *μ*M glycine (second panel), and after 30 minutes of washout (third panel). (b) Time course of 30 *μ*M NMDA and 10 *μ*M glycine effect on amplitude of the first pulse in each pair, with NMDA and glycine incubation at the time indicated by the bar. Data are mean ± s.e.m. from 5 cells. ^*∗*^ *P* = 0.0027, one-sample *t*-test. (c) Time course of 30 *μ*M NMDA and 10 *μ*M glycine effect on paired pulse ratio, with NMDA and glycine incubation at the time indicated by the bar. Data are mean ± s.e.m. from 5 cells.

**Figure 9 fig9:**
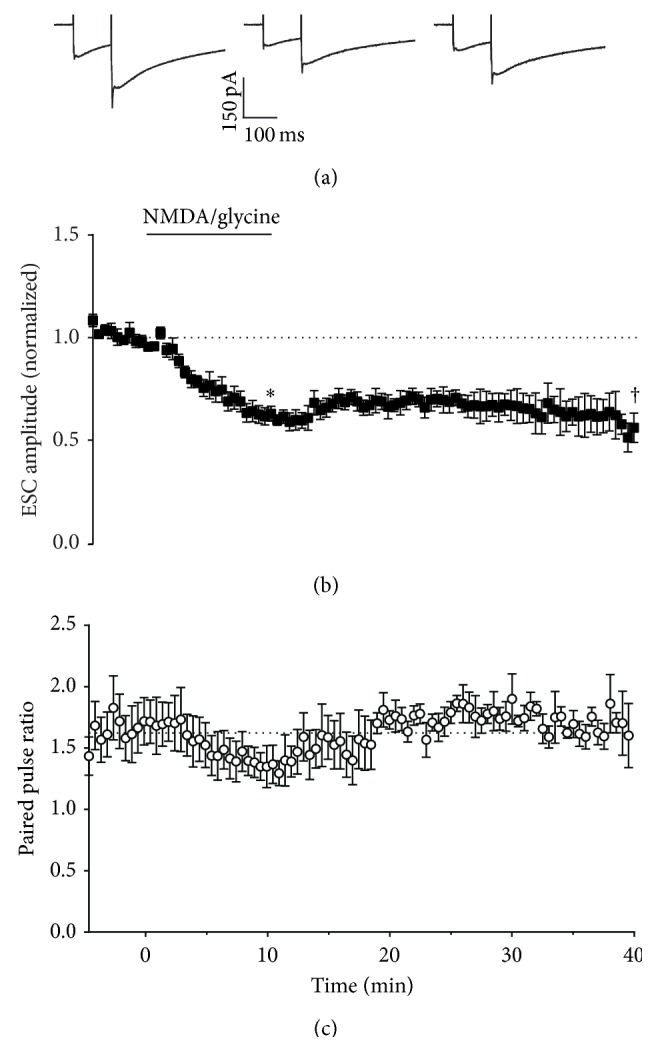
Effect of activation of NMDA receptors on Bergmann glial responses. (a) Representative whole-cell recordings of ESCs generated by paired pulse stimulation (100 ms interval) of parallel fibres in transverse cerebellar slices at 0.033 Hz before (first panel), after 10 minutes incubation with 30 *μ*M NMDA and 10 *μ*M glycine (second panel), and after 30 minutes of washout (third panel). (b) Time course of 30 *μ*M NMDA and 10 *μ*M glycine effect on amplitude of the first pulse in each pair, with NMDA and glycine incubation at the time indicated by the bar. Data are mean ± s.e.m. from 6 cells. ^*∗*^ *P* = 0.0003, ^†^ *P* = 0.0084, one-sample *t*-test. (c) Time course of 30 *μ*M NMDA and 10 *μ*M glycine effect on paired pulse ratio, with NMDA and glycine incubation at the time indicated by the bar. Data are mean ± s.e.m. from 6 cells.

**Figure 10:**
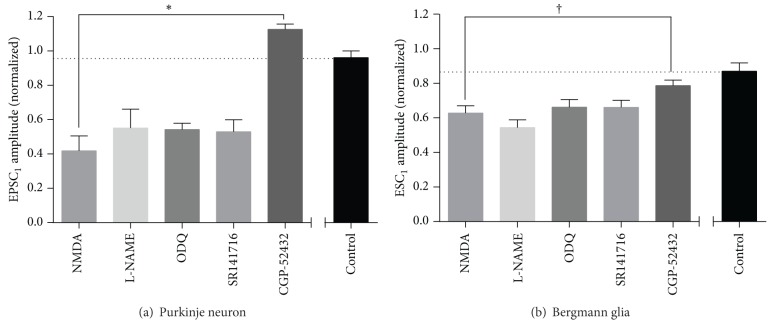
Modulation of NMDA-induced depression of neuronal and glial currents. (a) Mean EPSC_1_ amplitude following incubation with 30 *μ*M NMDA and 10 *μ*M glycine, either alone or in the presence of the stated inhibitors (L-NAME, 100 *μ*M; ODQ, 10 *μ*M; SR141716, 5 *μ*M; CPG 52432, 10 *μ*M). Control data from untreated cells stimulated for the same period are provided for reference. Data are mean ± s.e.m. from 5–7 cells. Statistical significance was tested by Dunnett's multiple comparisons test following one-way ANOVA. (b) Mean ESC_1_ amplitude following incubation with 30 *μ*M NMDA and 10 *μ*M glycine, either alone or in the presence of the stated inhibitors (L-NAME, 100 *μ*M; ODQ, 10 *μ*M; SR141716, 5 *μ*M; CPG 52432, 10 *μ*M). Control data are provided for reference. Data are mean ± s.e.m. from 5–9 cells. Statistical significance was tested by Dunnett's multiple comparisons test following one-way ANOVA. ^*∗*^ *P* < 0.0001; ^†^ *P* = 0.0105.

We therefore investigated the effect of NMDAR activation on synaptic and ectopic transmission. Under our experimental conditions, bath application of 30 *μ*M NMDA and 10 *μ*M glycine resulted in a reversible depression of synaptic transmission (Figures 8(a) [Fig fig9] and 8(b)), with an associated increase in paired pulse ratio (Figure 8(c)). We recorded for up to 40 min to determine whether synaptic depression could persist as reported by others [26] but always observed reversal. One small discrepancy between our protocol and others was the use of a sucrose-based buffer during slice preparation to improve survival of neurons. However, when experiments were repeated with slices prepared in standard saline-based cutting buffer, the results were indistinguishable (data not shown). In further contrast to previous reports, in our hands, inhibitors of NO-cGMP signalling were unreliable blockers of NMDA evoked depression (Figure 10(a)), with some cells giving depression of similar magnitude to control regardless of the presence of inhibitors. The mean depression in the presence of L-NAME, ODQ, and SR141716 was less than for NMDA alone, but given the variation from cell to cell, none of these interventions were statistically significant. In contrast, we found that preincubation with the GABA_B_R antagonist CGP 52432 completely blocked the synaptic depression evoked by NMDA/glycine (Figure 10(a)).

For ectopic transmission, NMDA/glycine caused a persistent inhibition that lasted after washout (Figures 9(a) and 9(b)) and a transient decrease in paired pulse ratio (Figure 9(c)). At ectopic sites, L-NAME, ODQ, and SR141716 were ineffective at blocking depression, but, as for synaptic sites, CGP 52432 significantly inhibited the depression (Figure 10(b)).

## 4. Discussion

Presynaptic LTP at the cerebellar parallel fibre synapse, induced by 4 Hz stimulation for 30 s, was first described by Salin et al. in 1996 [9]. Other reports corroborated these initial findings [10, 13, 21], and expanded the stimulation paradigms capable of evoking LTP to the 4–16 Hz range. The dominant mechanism of potentiation is activation of calcium/calmodulin activated adenylyl cyclase by the calcium influx resulting from action potential invasion of the terminal [12]. The resultant cAMP accumulation activates PKA and Epacs, leading to phosphorylation of several components of the vesicle release and priming apparatus [14, 28, 29], increasing release probability. The detailed molecular mechanism for LTP induction and maintenance is incompletely understood. For example, it is unclear whether cAMP signalling is sufficient for LTP, or whether nitric oxide signalling is also required.

In recent years, presynaptic LTD has joined presynaptic LTP as an established form of cerebellar plasticity [15, 16]. In slice preparations, LTD is revealed when PKA is inhibited [15], but in vivo, LTD appears to predominate with LTP being expressed only when LTD mechanisms are blocked [16]. For LTD, the concomitant activation of NMDA receptors and CB1 receptors is necessary, suggesting a multicellular mechanism, with activation of NMDA receptors on interneurons and endocannabinoid (probably 2-AG [22]) release from either interneurons or Purkinje neurons. Confounding interpretation further, postsynaptic LTD and presynaptic LTP have also been reported to depend on activation of interneuron NMDA receptors and nitric oxide synthesis [21, 26].

### 4.1. Presynaptic LTP at Synaptic but Not Ectopic Sites

In this study, we investigated how these presynaptic plasticity mechanisms affect neuron-glial transmission from parallel fibres to Bergmann glial cells. This connection depends on vesicular release of glutamate from sites outside of the presynaptic active zone, in a process termed ectopic release [1, 30]. Ectopic sites have similar short-term plasticity to synaptic sites but are also subject to depletion during repetitive stimulation, a phenomenon that means typical stimulation patterns for evoking postsynaptic plasticity result in depression of ectopic transmission [7]. Our goal was twofold: first to determine whether neuron-glial transmission showed similar patterns of presynaptic plasticity to synaptic transmission and second to thereby determine whether ectopic sites are likely to experience the same presynaptic signalling milieu as the active zone, which may help shed light on the anatomical localization of the ectopic pools. Our results suggest that ectopic sites do not express the presynaptic plasticity mechanisms present at the active zone and are instead functionally and anatomically independent.

Stimulation at 16 Hz for 15 s evoked presynaptic LTP of synaptic transmission (Figure 1), consistent with other reports [11]. In contrast, ectopic transmission expressed LTD in response to this stimulation pattern, but this depression did not appear to arise from activation of presynaptic inhibitory receptors. Instead, the likeliest explanation is that 16 Hz stimulation depleted a fraction of the ectopic pool that was not replenished by recycling mechanisms. This result suggests that activity-dependent activation of adenylyl cyclase did not generate a cAMP signal of sufficient amplitude or spatial range to engage downstream targets at ectopic sites. One possible explanation for this is limited distribution of R-type calcium channels, which may be positioned close to calcium-sensitive adenylyl cyclase in the active zone [31]. An alternative hypothesis is that the cAMP signal itself is spatially limited [32]. Interestingly, direct activation of adenylyl cyclase throughout the slice with bath application of forskolin induces potentiation at both synaptic and ectopic sites, but washout of forskolin leads to the rapid reversal of potentiation at ectopic sites, in contrast to synaptic sites that show sustained potentiation. This is consistent with the idea that localization of key aspects of the cAMP signalling apparatus to the active zone limits the focus of potentiation: a cAMP signal of sufficient amplitude or duration to “lock in” potentiation only occurs at synaptic release sites. An alternative hypothesis is that other mechanisms are required for maintenance or reinforcement of potentiation, and these mechanisms are absent from ectopic sites. Either way, the results suggest that cAMP synthesis primed by activity-dependent mechanisms is limited to the active zone, and LTP cannot be sustained at ectopic sites.

The effects of 16 Hz and forskolin treatment on paired pulse ratio of Bergmann glial ESCs were inconsistent with predictions for simple changes in release probability. A likely explanation for this outcome is the mixed nature of extrasynaptic currents in Bergmann glia, being composed of ionotropic AMPA receptor currents, electrogenic uptake of glutamate, and a slow current attributable to potassium uptake [5, 33, 34]. Variation in the relative amplitude of these different components between cells and after stimulation could lead to changes in ESC paired pulse ratio that are not necessarily due to presynaptic release probability alone.

### 4.2. Retrograde Inhibition by Endocannabinoids at Synaptic but Not Ectopic Sites

Direct activation of mGluR1 causes depression of parallel fibre to Purkinje neuron transmission [19]. Numerous studies have now established that mGluR1-dependent depression results from retrograde activation of CB1 receptors by 2-AG release [22]. CB1 receptor activation leads to inhibition of multiple isoforms of presynaptic calcium channels [35, 36], decreasing presynaptic release probability. This route for retrograde transmission has been implicated in numerous short-term and long-term forms of plasticity at the synapse, including, counterintuitively, postsynaptic LTP [37].

We previously reported that bath application of a CB1R agonist was able to depress neuron-glial transmission at ectopic sites [38], but the susceptibility of ectopic sites to cannabinoids synthesized and released in situ has not previously been investigated. Direct activation of mGluR1 with DHPG resulted in reversible depression of synaptic transmission, similar to previous reports [19, 20]. In contrast, no significant depression of ectopic transmission occurred during treatment (Figure 7; but note postwashout decrease in ESC), suggesting that endogenous 2-AG production did not engage CB1 receptors that were functionally coupled to ectopic release sites. Again, as with presynaptic LTP, this result can be explained by localization of the retrograde signal to the active zone, either by limitation of 2-AG diffusion or by a restricted distribution of CB1 receptors within the synaptic cleft. The former hypothesis seems to be most plausible, given that pharmacological activation of CB1R can depress ectopic release [38].

### 4.3. Activation of NMDA and GABA_B_ Receptors Depresses Both Synaptic and Ectopic Release

The involvement of presynaptic NMDA receptors in cerebellar plasticity has been an unexpected and contentious recent discovery. NMDAR-dependent synthesis of NO has been linked to postsynaptic LTD [26] and presynaptic LTP [10, 21]. Blockade of NMDA receptors also prevented presynaptic LTD, which was further dependent on CB1R activation [15]. The authors speculated that NMDAR activation on interneurons could lead to endocannabinoid release from the interneurons themselves, further complicating the crosstalk of signalling molecules between cells in the molecular layer.

Under our experimental conditions, direct activation of NMDA receptors by bath application of NMDA and glycine resulted in depression of both synaptic and ectopic transmission (Figures 8–10). In contrast to previous reports, inhibition of NO and CB1 signalling pathways did not reliably reverse the NMDAR depression. Mean depression was reduced by these interventions, but the cell to cell variability was so great that none of the reductions were statistically significant. Instead, we found that inhibition of GABA_B_ receptors fully reversed the NMDA/glycine effect. In contrast to presynaptic LTP or retrograde inhibition by mGluR1 activation, ectopic sites showed similar NMDA/glycine depression to synaptic sites. At ectopic sites NO and CB1 signalling inhibitors had no effect on depression, but again, a GABA_B_ receptor antagonist blocked it significantly.

These results are consistent with activation of NMDAR on molecular interneurons resulting in an increase in GABA release, such that tonic GABA rises to a level that activates GABA_B_ receptors throughout the presynaptic terminal to engage both synaptic and ectopic sites. This would be consistent with the reported heterosynaptic nature of GABA_B_R-dependent inhibition, where stimulation paradigms that trigger increased GABA release from interneurons cause widespread depression of parallel fibre synapses in the molecular layer [39].

It is not clear why this presynaptic mechanism predominates under our experimental conditions, but postsynaptic mechanisms predominate under other conditions [26, 27]. It is, however, noteworthy that the previous investigators routinely included a GABA_B_R antagonist in their bath solution. While it is surprising that this did not have so dramatic an effect on NMDA responses as we report, it would mean that the earlier investigations would by design have isolated only the postsynaptic consequences of NMDAR activation.

### 4.4. Signalling in and around the Synapse

**Figure 11:**
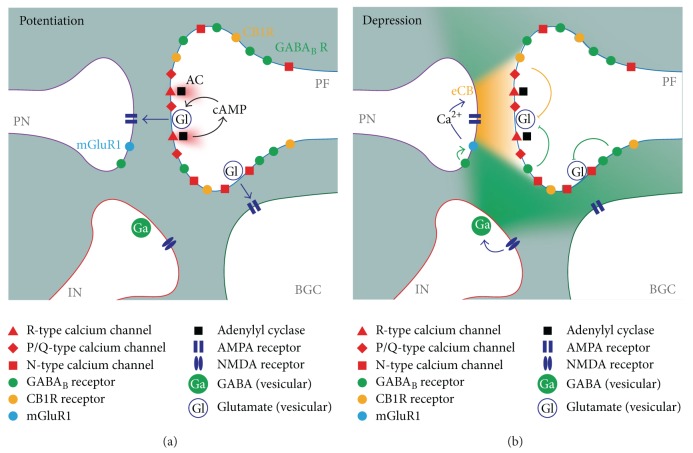
Localization of plasticity mechanisms at the parallel fibre terminal. (a) Illustration of the signalling domains involved in activity-dependent long-term potentiation. Local calcium influx through R-type channels activates adenylyl cyclase, leading to potentiation of vesicle release at the active zone. (b) Illustration of the signalling domains involved in presynaptic depression by CB1 receptors and GABA_B_ receptors. Activation of mGluR1 leads to local release of endocannabinoids in the active zone, possibly enhanced by postsynaptic GABA_B_R activation. Activation of NMDA receptors leads to widespread tonic GABA increases that engage both synaptic and ectopic release sites. Arrows denote activation mechanisms, bars denote inhibition. PN: Purkinje neuron, PF: parallel fibre, IN: interneuron, and BGC: Bergmann glial cell. Synaptic sites in active zone activate PN AMPA receptors, and ectopic sites in terminal periphery activate BGC AMPA receptors.

Although there is considerable overlap between the signalling pathways involved in presynaptic and postsynaptic mechanisms for plasticity (with many requiring NMDAR, CB1R, and NO signalling), meaning that the predominant effect is variable with individual experimental conditions, our evidence suggests that ectopic sites do not follow the same activity-dependent rules for induction and maintenance of either presynaptic LTP or presynaptic LTD as active zones. The most straightforward explanation for this effect is spatial limitation of the signalling events required for induction and maintenance of plasticity.  Figure 11 illustrates this effect, with LTP and LTD mechanisms restricted to the active zone, but diffusive inhibition via tonic GABA engaging both release sites.

Collectively, these findings reinforce the concept of independent operation of synaptic and ectopic sites, despite being located within the same presynaptic cell and possibly even the same bouton [2]. This further emphasizes the functional independence of neuron-glial transmission and synaptic transmission, meaning that alterations in the strength of transmission to the different cell classes can be governed by different mechanisms and follow different computational rules.
